# Variations of en Bloc Resection for Advanced External Auditory Canal Squamous Cell Carcinoma: Detailed Anatomical Considerations

**DOI:** 10.3390/cancers13184556

**Published:** 2021-09-10

**Authors:** Noritaka Komune, Daisuke Kuga, Koichi Miki, Takashi Nakagawa

**Affiliations:** 1 Department of Otorhinolaryngology, Graduate School of Medical Sciences, Kyushu University, Fukuoka 812-8582, Japan; nakataka@med.kyushu-u.ac.jp; 2 Department of Neurosurgery, Graduate School of Medical Sciences, Kyushu University, Fukuoka 812-8582, Japan; kuga@ns.med.kyushu-u.ac.jp; 3 Department of Neurosurgery, Fukuoka University Hospital and School of Medicine, Fukuoka 814-0180, Japan; em.koichi@gmail.com

**Keywords:** external auditory canal, squamous cell carcinoma, temporal bone resection, surgical anatomy

## Abstract

**Simple Summary:**

From the viewpoint of surgical anatomy, surgical patterns of temporal bone cutting to achieve negative margin resection for advanced squamous cell carcinoma of the external auditory canal can be divided into four categories: conventional lateral temporal bone resection (LTBR), extended LTBR, conventional subtotal temporal bone resection (STBR), and modified STBR. Extended LTBR is divided into four types: superior, inferior, anterior, and posterior procedures. Several directional extension procedures can be combined based on the extension of the tumor to achieve negative margin resection. Furthermore, en bloc resection with the temporomandibular joint or glenoid fossa increases the technical difficulty of a surgical procedure because the exposure and manipulation of the petrous segment of the internal carotid artery are limited from the middle cranial fossa. Accurate preoperative evaluation of the tumor extension and preoperative consideration of the exact resection line are required to achieve negative margin resection.

**Abstract:**

Currently, only lateral temporal bone resection (LTBR) and subtotal temporal bone resection (STBR) are widely utilized for the surgical treatment of advanced squamous cell carcinoma of the external auditory canal (EAC-SCC). However, there are few descriptions of variations on these surgical approaches. This study aimed to elucidate the variations of en bloc resection for advanced EAC-SCC. We dissected the four sides of cadaveric heads to reveal the anatomical structures related to temporal bone resection. From the viewpoint of surgical anatomy, surgical patterns of temporal bone cutting can be divided into four categories: conventional LTBR, extended LTBR, conventional STBR, and modified STBR. Extended LTBR is divided into four types: superior, inferior, anterior, and posterior extensions. Several extension procedures can be combined based on the extension of the tumor. Furthermore, en bloc resection with the temporomandibular joint or glenoid fossa increases the technical difficulty of a surgical procedure because the exposure and manipulation of the petrous segment of the internal carotid artery are limited from the middle cranial fossa. Surgical approaches for advanced SCC of the temporal bone are diverse. They require accurate preoperative evaluation of the tumor extension and preoperative consideration of the exact line of resection to achieve marginal negative resection.

## 1. Introduction

Currently, only lateral temporal bone resection (LTBR) and subtotal temporal bone resection (STBR) are widely used for the surgical treatment of advanced squamous cell carcinoma of the external auditory canal (EAC-SCC). However, there are few descriptions of variations to these surgical approaches [1]. Furthermore, several challenges with regard to the surgical approach for advanced EAC-SCC need to be overcome.

The first challenge is to determine whether piecemeal or en bloc resection improves the prognosis [2,3,4,5,6,7,8,9,10,11,12,13,14]. Campbell et al. and Ward et al. first attempted to apply the concept of en bloc resection beyond the usual radical mastoidectomy in 1951 [5,6]. In 1954, Persons and Lewis officially introduced en bloc resection of the temporal bone [7]. After its introduction, several groups made further advances to this challenging procedure [8,9,10,11,12,13]. To safely achieve en bloc resection, in 1981 Ariyan et al. emphasized the importance of an interdisciplinary surgical team, formed by neurosurgeons, otolaryngologists, and plastic surgeons, for the surgical treatment of this highly lethal type of cancer [14]. Nowadays, en bloc resection appears to be more acceptable than piecemeal resection from the oncological viewpoint; nevertheless, this topic remains under debate.

Another challenge is the lack of guidelines on the selection of a surgical approach for en bloc resection and its contraindications. Classically, LTBR and STBR have been used for early- and advanced-stage EAC-SCC, respectively. However, this has led to misconceptions regarding the applications of en bloc surgery. Apart from early-stage temporal bone-SCC, LTBR can also be applied to advanced-stage EAC-SCC. However, depending on the direction of the extension of the advanced tumor, conventional LTBR (cLTBR) can be insufficient to achieve en bloc resection with a negative margin, thereby compromising the oncologic principle of en bloc resection. Depending on the direction of tumor extension, the surgical procedure and technical difficulty differ significantly.

The suitability of cLTBR for en bloc resection of EAC-SCC is widely recognized. This procedure can be performed at any institution and uses a consistent surgical technique. However, if the tumor extends beyond the range of cLTBR, a detailed anatomy-based description of the variations of surgical procedure is rarely provided. In this study, variations of en bloc resection for advanced EAC-SCC were investigated in detail based on cadaveric dissection and a previous literature review.

## 2. Materials and Methods

### 2.1. Cadaveric Dissection

To reveal the anatomical structures related to temporal bone resection in advanced EAC-SCC, four sides of formalin-perfused adult cadaveric heads, in which arteries and veins were injected with red- or blue-colored silicone rubber (Dow Corning Corp., Midland, MI, USA), were dissected. Cadaveric dissection was performed by first author (N.K.).

### 2.2. Literature Review

MEDLINE (1950–2021) searches were conducted using the keywords “external auditory canal”, “external auditory meatus”, “middle ear”, or “temporal bone”, with the term “resection” in the title or abstract. The selected publications were limited to English literature focused on surgical techniques for en bloc temporal bone resection. Furthermore, we searched the reference list of each article for other reports on en bloc temporal bone resection that may have been missed in our initial MEDLINE searches. Subsequently, we selected publications that included detailed descriptions of the surgical techniques.

### 2.3. Treatment Strategy

Our treatment policy for advanced EAC-SCC was as follows. The main treatment strategy was to perform surgery for all resectable cases. Surgical procedures were determined based on the direction of the tumor extension and included cLTBR, extended LTBR (eLTBR), modified STBR (mSTBR), and conventional STBR (cSTBR). Among advanced-stage cases (T3 and T4 on the Pittsburgh classification), we selected the appropriate approach when the tumor shrank sufficiently for resection. Contraindication of surgical intervention was considered when the tumor invasion extended to the internal carotid artery, dura, brain parenchyma, cavernous sinus, nasopharynx, or petrous apex medial to the otic capsule. Chemoradiotherapy was also selected for patients that could not be treated by or refused radical surgery. If resectable, residual lesions after curative radiotherapy (RT) were surgically removed. RT was administered five days per week (1.6–2.0 Gy/fraction, for a total dose of 60–70 Gy) accompanying triweekly cisplatin (100 mg/m^2^, once every three weeks, 2–3 cycles). The TPF (docetaxel, cisplatin, fluorouracil) regimen was used as induction chemotherapy (5-fluorouracil: 600 mg/m^2^, days 1–5; cisplatin: 60 mg/m^2^/day, day 1; docetaxel: 60 mg/m^2^, day 1) once every three weeks (1–2 cycles).

### 2.4. Case Profiles

We retrospectively reviewed the surgical cases with en bloc temporal bone resection for advanced EAC-SCC at our institution from October in 2016 to March in 2021 and examined the variations of surgical procedures for en bloc temporal bone resection. All procedures were performed by the first author (N.K.) and supervised by the last author (T.N.).

### 2.5. Statistical Analysis

For all recorded data, statistical analyses were performed using JMP 6.1 software (SAS Institute, Cary, NC, USA). The survival rate was calculated using the Kaplan–Meier method. The influence of the margin status after tumor resection on the overall survival of patients was calculated using a log-rank test. *p*-values < 0.05 denoted statistically significant differences.

## 3. Results

Initially, we performed cadaveric dissection to reveal the surgical anatomy related to en bloc temporal bone resection for advanced EAC-SCC.

### 3.1. Anatomical Considerations

#### 3.1.1. Relationship between the Glenoid Fossa and Petrous Segment of the Internal Carotid Artery

**Figure 1:**
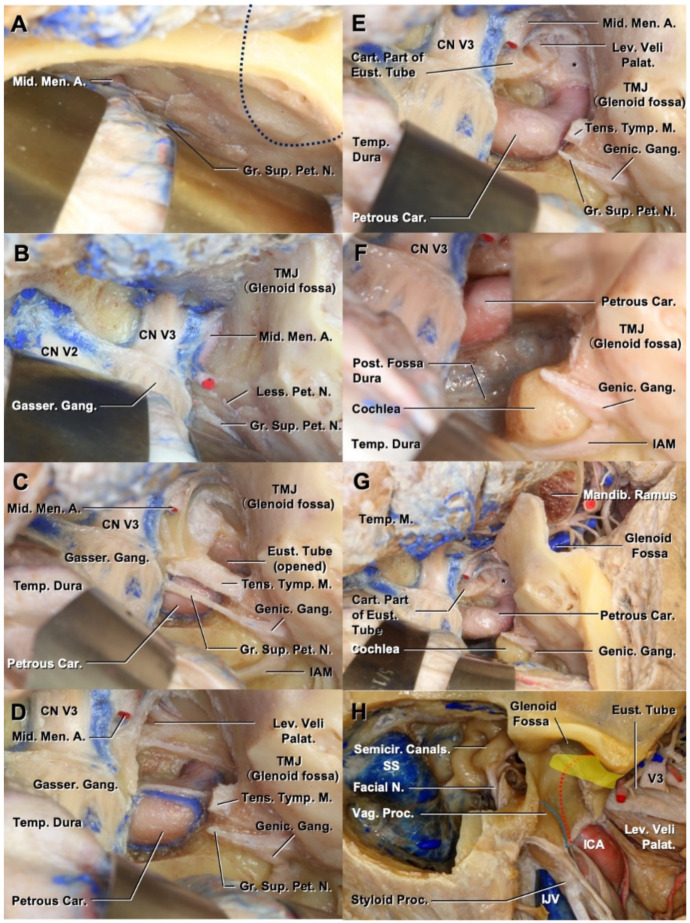
Relationship between the glenoid fossa and petrous carotid. (**A**) Temporal dura is elevated and the middle meningeal artery is identified. The blue dotted line indicates the position of the glenoid fossa/temporomandibular joint. (**B**) Middle cranial base is drilled to expose the course of the middle meningeal artery and CN V3. (**C**,**D**) Bone is drilled between the foramen ovale and glenoid fossa to expose the course of the petrous carotid. (**E**) After opening the carotid canal, the fibrocartilaginous tissue (*) is exposed. (**F**) Anterior translocation of the petrous carotid. (**G**) Condyle of the mandible is removed to investigate the relationship between the glenoid fossa and petrous carotid (asterisk indicates the fibrocartilaginous tissue). (**H**) The relationship between the vaginal process of the tympanic part of the temporal bone and the petrous carotid is depicted. The red dotted line indicates the course of the petrous carotid medial to the vaginal process. The blue dotted line shows the course of the jugular bulb. The yellow area shows the course of the eustachian tube lateral to the petrous carotid. A., artery; Car., carotid; CN, cranial nerves; Eust., eustachian tube; Gang., ganglion; Gasser., gasserian; Genic., geniculate; Gr., greater; IAM, internal auditory meatus; L, lateral semicircular canal; Less., lesser; Lev., levator; M., muscle; Mandib., mandibular; Men., meningeal; Mid., middle; N., nerve; Sup., superficial; Palat., palatine; Pet., petrosal; Post., posterior; Proc., process; Semicir., semicircular; SS, sigmoid sinus; Temp., temporal; Tens., tensor; TMJ, temporomandibular joint; Tymp., tympanic; Vag., vaginal.

We drilled the middle fossa floor to reveal the relationship between the glenoid fossa and the petrous segment of the internal carotid artery (petrous carotid). The dura of the middle fossa was elevated to expose the middle meningeal artery and greater petrosal nerve (Figure 1A). Dividing the middle meningeal artery and splitting the dura over V2 and V3 allowed the elevation of the dura medially. The foramen ovale was opened by drilling the middle cranial base while preserving the glenoid fossa (Figure 1B). The bone between the glenoid fossa and foramen ovale was drilled to expose the eustachian tube, roofed with the tensor tympani muscle (Figure 1C). The carotid canal was located immediately lateral to the bony portion of the eustachian tube and ran parallel to the superficial greater petrosal nerve. After cutting the tensor tympani and greater petrosal nerve and drilling the bone superior and lateral to the horizontal and vertical portion of the petrous carotid, the course of the petrous carotid was exposed from the vertical to the horizontal segment (Figure 1D). The entrance of the carotid canal was surrounded by a fibrocartilaginous ring (Figure 1E). The levator veli palatine muscle was inferiorly attached to the cartilaginous eustachian tube (Figure 1E). After cutting the fibrocartilaginous ring around the artery at the entry in the carotid canal, the artery was translocated anteriorly to provide space for drilling the bone medial to the artery (Figure 1F). Finally, the mandibular condyle was removed to investigate the relationship between the glenoid fossa and petrous carotid (Figure 1G). If the glenoid fossa/temporomandibular joint (TMJ) needs to be resected en bloc with the EAC so it prevents exposure of the tumor, the petrous carotid needs to be controlled from the middle cranial fossa floor, rather than the glenoid fossa. When the glenoid fossa is exposed and it does not lead the exposure of cancer, the carotid canal can be exposed by drilling the vaginal process of the temporal bone through the glenoid fossa. The relationship between the vaginal process and petrous carotid is illustrated in Figure 1H.

#### 3.1.2. Access to the Jugular Foramen

**Figure 2:**
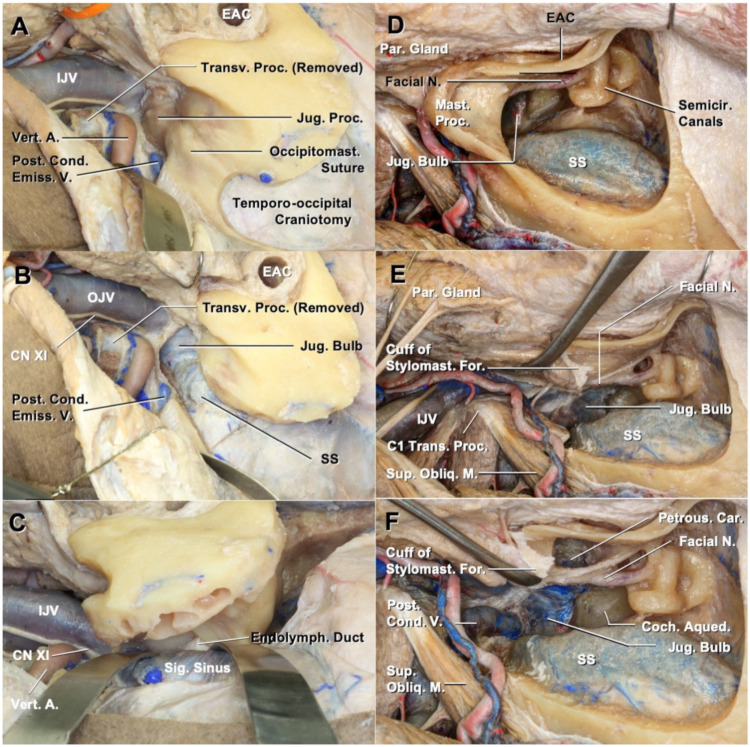
Two approaches to opening the jugular bulb. (**A**) The view after high cervical exposure and temporo-occipital craniotomy. (**B**) Removal of the jugular process is performed to expose the jugular bulb. (**C**) Sigmoid sinus and jugular bulb can be separated from the temporal bone. (**D**) Jugular bulb is exposed after mastoidectomy. High cervical exposure is completed. (**E**) Removal of the mastoid process and jugular process widens the exposure of the jugular bulb. (**F**) Fallopian bridge technique exposes the petrous carotid, preserving the posterior wall of the external auditory canal. A., artery; Car., carotid; Cond., condylar; EAC, external auditory canal; Emiss., emissary; Endolymph., endolymphatic; For., foramen; IJV, internal jugular vein; N., nerve; Obliq., oblique; Par., parotid; Post., posterior; Proc., process; SS, sigmoid sinus; Stylomast., stylomastoid; Sup., superior; V., vein; Vert., vertebral.

There are two types of exposure of the jugular foramen. Large temporo-occipital craniotomy was performed, and the inferior surface of the jugular process of the occipital bone was exposed (Figure 2A). The jugular process was drilled to expose the sigmoid sinus and jugular bulb (Figure 2B). The sigmoid sinus and dura of the posterior fossa were separated from the posterior surface of the temporal bone (Figure 2C). After cutting the endolymphatic sac and cranial nerves (CNs) VII and VIII, the elevation of the dura was proceeded to the jugular fossa.

The transmastoid approach to the jugular foramen was applied in the eLTBR or mSTBR. The bone inferior to the posterior semicircular canal, while following the posterior fossa dura, was drilled to reach the jugular foramen (Figure 2D). The mastoid tip and lateral part of the jugular process were removed to widen the surgical view around the jugular bulb (Figure 2E). The carotid canal was exposed through the mastoid and tympanic cavity, and we could drill the bone, while the root of the styloid process was attached to the vaginal process (Figure 2F).

#### 3.1.3. Manipulation of Facial Layers Attached to the Skull Base around the Vaginal Process

**Figure 3:**
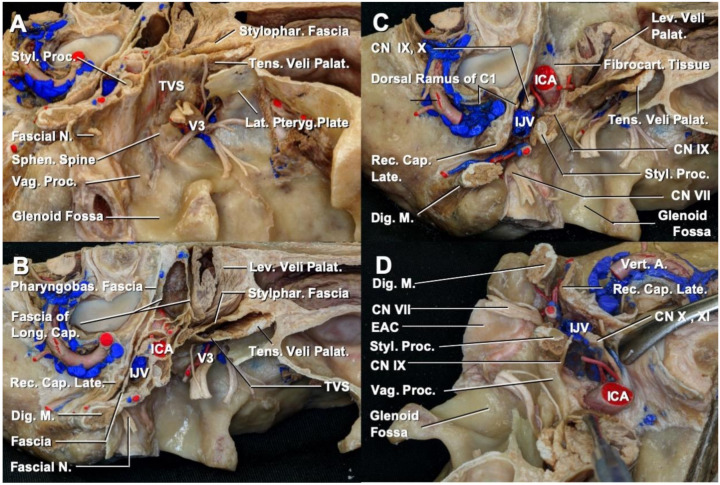
Fascial tissue attached around the vaginal process. (**A**) Inferolateral view. The fascial layers attached to the vaginal process are shown in the right cadaveric head. Tensor vascular styloid fascia forms a part of the carotid sheath. (**B**) Inferior view. The carotid sheath was composed of the stylopharyngeal fascia, tensor vascular styloid fascia, pharyngobasilar fascia, fasciae of the longus capitis, and fascia anterior to the rectus capitis lateralis. (**C**) Inferior view after removal of the carotid sheath. (**D**) Anteroinferior view. The glossopharyngeal nerve coursing medially to the root of the styloid process and vaginal process. A., artery; C.N., cranial nerve; Cap., capitis; Dig., digastric; EAC, external auditory canal; Fibrocart., fibrocartilaginous; ICA, internal carotid artery; IJV, internal jugular vein; Lat., lateral; Late., lateralis; Lev., levator; Long., longus; N., nerve; Palat., palatini; Pharyngobas., pharyngobasilar; Proc., process; Pteryg., pterygoid; Rec., rectus; Sphen., sphenoid; Stylophar., stylopharyngeal; Styl., styloid; Tens., tensor; TVS, tensor-vascular-styloid fascia; Vert., vertebral; Vag., vaginal.

Dealing with the soft tissue attached to the skull base is an essential factor for achieving negative margin resection. The fascia around the carotid canal, jugular foramen, and vaginal process of the temporal bone are shown in Figure 3. The tensor-vascular styloid and stylopharyngeal fasciae were fused and attached immediately medial to the sphenoid spine and lateral surface of the vaginal process of the temporal bone covering the styloid apparatus (Figure 3A,B). If the tumor extends inferiorly and approaches the jugular foramen, the styloid process should be resected en bloc with the EAC, while enclosing the tumor by the tensor vascular styloid fascia (Figure 3A,B).

Immediately below the jugular foramen and carotid canal, glossopharyngeal nerve (CN IX) runs more anteriorly, immediately medial to the stylopharyngeal muscle (Figure 3C,D). When dissecting the fascial layer attached to the skull base, greater caution should be exercised to avoid damaging CN IX than CN X or XI.

### 3.2. Variation of Bone Cutting for en Bloc Temporal Bone Resection

**Figure 4:**
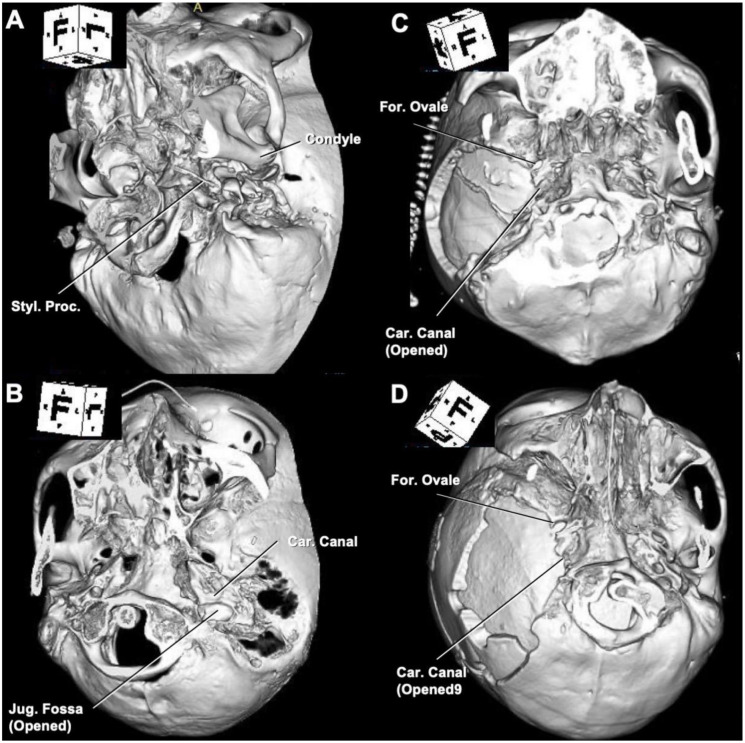
Three-dimensional (3D) bone reconstruction after temporal bone resection. (**A**) Conventional lateral temporal bone resection (representative case of cT2). (**B**) Lateral temporal bone resection with anterior and posterior extension (case 8); (**C**) Modified subtotal temporal bone resection (case 13). (**D**) Conventional subtotal temporal bone resection en bloc with TMJ (case 15). 3D, three-dimensional; Car., carotid; Jug., jugular; Proc., process; Styl., styloid; TMJ, temporomandibular joint.

**Figure 5:**
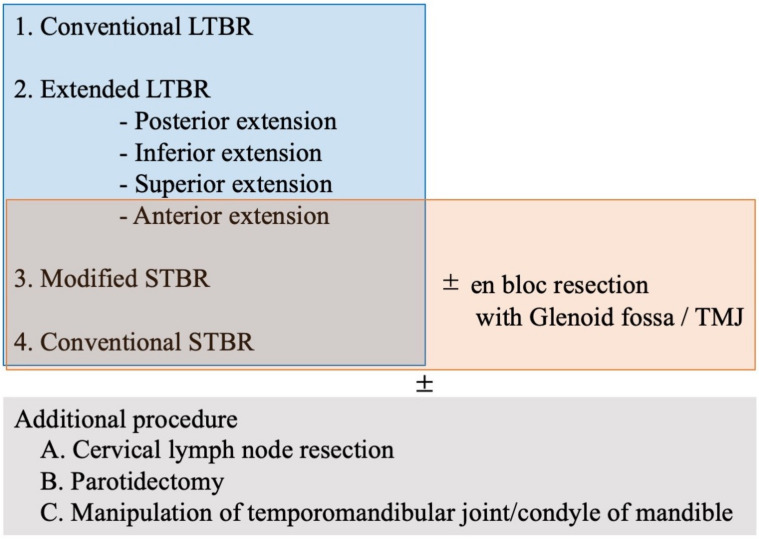
Variation of temporal bone resection. LTBR, lateral temporal bone resection; STBR, subtotal temporal bone resection; TMJ, temporomandibular joint.

**Figure 6:**
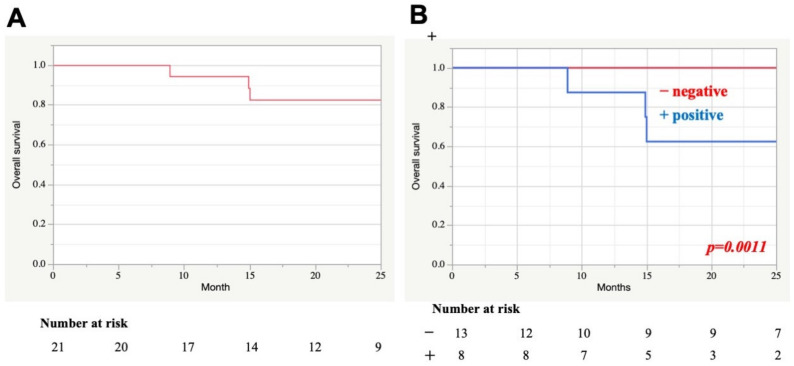
Kaplan–Meier curves for 2-year overall survival. (**A**) The 2-year overall survival after temporal bone resection in all cases. (**B**) Two groups are compared: cases with negative and positive margin resection.

The range of osteotomy differs between procedures. In cLTBR, osteotomy was limited as shown in Figure 4A. However, if the tumor extended anteriorly, inferiorly, superiorly, and posteriorly from the EAC, it was impossible to remove the tumor with a negative margin using cLTBR. We applied eLTBR if the tumor extended inferiorly and was close to the jugular foramen and the styloid process, which was resected en bloc with the EAC; the opening of the jugular foramen was often required to complete the tumor resection with a negative margin (Figure 4B). If the tumor extended into the middle ear, STBR was necessary. If the invasion of the tumor into mastoid cavity was limited, mSTBR, (Figure 4C) combined with posteriorly limited mastoidectomy and temporal craniotomy, was sufficient to complete the en bloc resection. However, if the tumor extended to the mastoid cavity and middle ear, we needed to perform cSTBR, including retromastoid-paracondylar approaches and large temporo-occipital craniotomy (Figure 4D). From the perspective of surgical anatomy, temporal bone cutting can be divided into several patterns (Figure 5 and Figure 6) Whether the petrous carotid can be exposed through the glenoid fossa (transglenoid fossa procedure: TGP) could affect the difficulty of the exposure and translocation of the petrous carotid (Figure 5).

### 3.3. Case Profile

The profiles of the 21 patients included in the study are summarized in Table 1. Our dataset included six males and 15 females (median age: 66 years; range: 33–83 years). The median follow-up interval was 24.7 months (range: 4.7–71 months). The clinical stages of the cases included T3 (*n* = 10, 48%) and T4 (*n* = 11, 52%). Two of 11 T4 cases were recurrent cases. Of the 21 patients, four developed cervical lymph node metastasis (19%). There were no patients with distant metastasis. Well-differentiated SCC was observed in 71% of the cases.

Detailed information regarding the surgical procedures is shown in Table 2. Of note, cLTBR was applied to nine of 21 cases (43%). For the other cases, eLTBR or STBR were required to achieve en bloc resection of the tumor. However, lower cranial nerve palsy was not found postoperatively. The 2-year overall survival rate for all cases was 82.6% (Figure 6A). En bloc resection with a negative margin significantly improved the patient prognosis (*p* = 0.0011) (Figure 6B).

## 4. Discussion

### 4.1. Surgical Strategy for Advanced EAC-SCC

Conventionally, malignant tumors of the head and neck are treated with en bloc tumor resection to the set safety margins. Zanoletti et al. reported that recurrences occurred despite obtaining block resection with negative margin in EAC-SCC [15], but many previous studies concluded that negative margin resection can improve the prognosis of EAC-SCC [16,17,18,19,20,21]. It may be difficult to control positive margin resection with adjuvant therapy [22,23,24,25], although some studies recommended a piecemeal approach for cases requiring treatment beyond LTBR due to the technical difficulty [2,3,4,26]. Technically, the surgical approach is more demanding than the piecemeal approach for en bloc resection. However, postoperative pathological examination of the margin can be performed more objectively with the former approach. Although piecemeal resection is safer, the results of the margins may be influenced by the surgeon’s subjective judgment during surgery. Moreover, postoperative negative margins cannot be determined pathologically. The results of the margin evaluation depend on the subjective judgment of the surgeon. The treatment of advanced EAC-SCC requires a multidisciplinary approach. Rapid postoperative evaluation of the margin and determination of the necessity of adjuvant chemoradiotherapy are crucial. From this perspective, the importance of en bloc resection rather than piecemeal resection can be recognized. Furthermore, the current multidisciplinary strategy for the treatment of EAC-SCC reduces morbidity and mortality even among those undergoing surgery for advanced disease, such as cSTBR.

### 4.2. Surgical Options for Advanced EAC-SCC

#### 4.2.1. Concept of en Bloc Temporal Bone Resection

LTBR is an otologic procedure used for the en bloc removal of the EAC along with the tympanic membrane. However, STBR requires a multidisciplinary approach. The procedure involves en bloc removal of the temporal bone, including (or transecting) the otic capsule. En bloc total temporal bone resection (i.e., en bloc removal including the most apical part of the petrous apex) is theoretically impossible without resection of the petrous carotid [27,28]. LTBR varies from the cLTBR to the eLTBR, including the anterior, inferior, superior, and posterior extensions (Figure 5). In addition to cSTBR, the modification of the STBR has been reported to reduce the invasiveness and the incidence of complications (Figure 5).

#### 4.2.2. Variation of the Lateral Skull Base Bone Cutting

**Figure 7:**
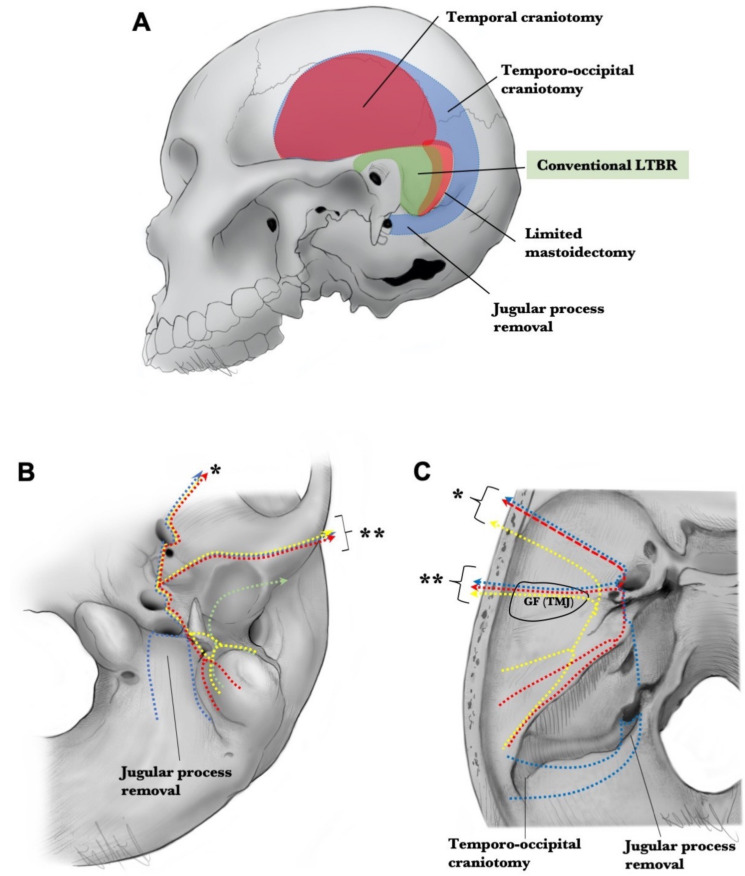
Variation of the cutting line for temporal bone resection. (**A**) Variation of craniotomy for temporal bone resection. (**B**,**C**) A cutting line on the inferior (**B**) and superior surface (**C**) of the skull base. The green dotted line shows the cutting line for conventional lateral temporal bone resection. The yellow dotted line indicates the cutting line for the extended LTBR. The red dotted line shows the modified subtotal temporal bone resection. The blue dotted line indicates the cutting line for the conventional subtotal temporal bone resection. Single asterisk shows the cut line for the en bloc resection with glenoid fossa/TMJ. Double asterisks show the transglenoid fossa procedure. LTBR, lateral temporal bone resection; TMJ, temporomandibular joint.

In both T3 and T4 cases, the limits of tumor extension should be accurately assessed preoperatively through thin-slice contrast-enhanced computed tomography and contrast-enhanced magnetic resonance imaging [29]. The bone cutting line can then be carefully considered to achieve negative margin resection (Figure 7).

### 4.3. Conventional LTBR

In summary, a cortical mastoidectomy is performed and extends anteriorly superior to the EAC into the glenoid fossa. Next, extended posterior tympanotomy is completed, and the tympanic part of the temporal bone and its vaginal process is cut forward, while the inferior bony wall of the EAC is preserved. Next, anterior and inferior cutting lines are connected using an osteotome or surgical drill (Figure 7A,B). This procedure is well-established and sophisticated and can be completed by a single otologist. In addition, it can be applied to some early T3 and T4 cases without tympanic cavity extension.

### 4.4. Extended LTBR

This extended procedure can be applied to advanced T3 or T4 cases without tympanic cavity extension. Of note, the procedure of temporal bone cutting differs depending on the direction of the tumor extension. Several extension directions can be used in combination for cases where this is necessary.

#### 4.4.1. Inferior Extension

In case of potential invasion of the stylomastoid foramen, which causes facial palsy, bone cutting is set medial to the stylomastoid foramen while cutting the vertical segment of the facial nerve. The tumor will likely be enclosed in the tensor vascular styloid fascia (Figure 7B). This procedure often requires opening the jugular foramen after the infralabyrinthine mastoidectomy. The root of the styloid process is extremely close to the jugular foramen. Thus, en bloc resection just lateral to the venous wall of the jugular bulb and internal jugular vein is often necessary to ensure the safety margin (Figure 7B). The glossopharyngeal nerve runs immediately medially to the root of the styloid process. Care should be taken to avoid injuring the glossopharyngeal nerve during the manipulation of the fascial tissue attached to the styloid process or vaginal process of the tympanic part of the temporal bone (Figure 3C,D).

#### 4.4.2. Superior Extension

When the tumor extends superiorly, temporal craniotomy is required (Figure 6A). If the tumor does not invade the temporal lobe of the cerebrum, the superior cut is performed from the middle cranial base. The cutting line extends from the epitympanic cavity and tympanic ostium of the eustachian tube to the glenoid fossa and connects the inferior bone cutting line (Figure 7B,C). En bloc resection with dura matter remains debatable, and Nakagawa et al. have stated that posterior cranial dural invasion is a contraindication for surgery, but surgery is considered for cases with dural invasion to the middle cranial fossa [20].

#### 4.4.3. Posterior Extension

Tumors in the EAC can often destroy the posterior wall of the canal into the mastoid cavity. This extension of the tumor easily reaches the vertical segment of the facial nerve. There is insufficient space between the tumor and the vertical segment of the facial nerve. To achieve the negative margin resection, facial nerve resection needs to be considered (Figure 7B). In such a case, retrofacial mastoidectomy needs to be performed in the tympanic cavity. If mastoid air cell opacification is identified widely due to the extensive invasion, eLTBR is not a suitable procedure from an oncological viewpoint.

#### 4.4.4. Anterior Extension

Tumors with anterior extension occasionally require en bloc resection with the glenoid fossa/TMJ (Figure 5). When the tumor extends anteriorly but does not invade the condylar process or bone of the glenoid fossa, the surgeon can separately remove the condylar process and proceed with tumor removal, confirming the tumor margin. If the bone of the condylar process has been invaded by the tumor but the glenoid fossa has not, the neck of the condyle should be cut, the condyle translocated laterally, and the periosteum separated from the bone of the glenoid fossa. This can expose the bony surface of the vaginal process rendering en bloc resection with TMJ possible. In such situations, the petrous carotid can be exposed by drilling the bone through the glenoid fossa. In case of invasion to the bone of the glenoid fossa or TMJ, which does not allow the exposure of the vaginal process preventing the exposure of the tumor, the surgical procedure is completely different because temporal craniotomy is required. The superior cut is performed from the epitympanic cavity and tympanic ostium of the eustachian tube to the greater wing of the sphenoid bone, while passing laterally to the foramen spinosum and anterior to the glenoid fossa and connecting the anterior and inferior bone cutting line (Figure 7B,C) [30]. This procedure enables complete en bloc resection with the TMJ/glenoid fossa.

### 4.5. Conventional STBR

Conventional STBR requires temporo-occipital craniotomy (Figure 7A) and removal of the condyle after cutting the ramus of the mandible or neck of the process. Dura of the middle fossa and posterior fossa from the mastoid and petrous bone are separated from the temporal bone. The petrous carotid can be exposed through the glenoid fossa. The internal auditory meatus can be cut either from the middle cranial fossa or posterior cranial fossa. Finally, a medial cut of the pyramidal bone can be achieved immediately lateral to the vertical segment of the petrous carotid (Figure 7B,C).

Exposure of the vertical to horizontal segments of the petrous carotid from the glenoid fossa has been reported with clear illustrations from two research groups [11,14]. Ariyan et al. described that, after the removal of the condylar process, through the glenoid fossa, the drilling of the vaginal process of the tympanic part of the temporal bone exposes the chorda tympani in the petrotympanic fissure, tensor tympani, and eustachian tube, and these structures are cut [14]. The anterior aspect of the petrous carotid from the horizontal segment to the vertical segment is then exposed.

In case of invasion to the bone of the glenoid fossa/TMJ, surgical procedures are completely different because it is not possible to control the petrous carotid through the glenoid fossa. In such a case, en bloc resection with the glenoid fossa is necessary, and the petrous carotid needs to be exposed and manipulated from the middle cranial fossa floor (Figure 5). We previously reported the combination of cSTBR with glenoid fossa in step-by-step manner [31,32]. This is composed of three approaches, namely high cervical, subtemporal-infratemporal fossa, and retromastoid paracondylar.

Manipulation of the internal auditory meatus, which includes CN VII and VIII, can be performed through the middle cranial fossa or posterior cranial fossa based on the extent of tumor infiltration. The method of final bony cut for en bloc resection has been reported. The final cut is performed with a microsurgical technique using a high-speed drill [7,33], chisel [1,14], or diamond thread wire saw [34]. We prefer to use the diamond bar with microsurgical technique to complete the final cut. When the temporal bone became mobile, the venous wall of the jugular bulb is separated from the jugular fossa. The soft tissue attached to the skull base around the jugular foramen and carotid canal are dissected, avoiding injury to the main vessels and lower cranial nerves, especially the glossopharyngeal nerve. If the tumor extends close to the jugular foramen or carotid artery, it should be resected with the fascia, including the tensor vascular styloid fascia and carotid sheath, thereby preventing tumor exposure.

### 4.6. Modified STBR

The surgical step of cSTBR varies among institutions because the surgical procedure is highly challenging and has not been well-established due to the rarity of this form of cancer. To reduce the morbidity or mortality, Nakagawa et al. [20] reported a modified STBR, which includes temporal craniotomy rather than temporooccipital craniotomy, and limited posterior mastoidectomy (Figure 7B,C). This method does not require a retromastoid paracondylar approach and, instead, involves a limited posterior mastoidectomy. The limited posterior mastoidectomy enabled us to cut the internal auditory meatus and expose the jugular foramen from the lateral aspect [16]. However, there is a debate with the mSTBR approach. In case of invasion of the mastoid cavity by the tumor, which would result in mastoid opacification, the opening of the cavity can prevent the surgeon from achieving a negative resection margin. Nakagawa’s group achieved negative margin resection in 10 of 13 patients who underwent mSTBR [16]. They administered preoperative chemoradiotherapy, which possibly limited the ability of micrometastatic cells to proliferate in the cavity [16]. Currently, there is no evidence that opening a fluid-filled mastoid cavity worsens patient outcomes. Posterior limited mastoidectomy is more familiar to otologists than the retromastoid paracondylar approach. This modified procedure is a less invasive option for complete en bloc resection of the temporal bone.

### 4.7. Additional Procedures

#### 4.7.1. Cervical Lymph Node Dissection

In addition to temporal bone cutting, the extent of the cervical lymph node dissection should be considered according to the clinical stage of the cases. However, currently, there are no guidelines for cervical lymph node dissection [35,36]. In 2020, Kiyokawa et al. reported that levels Ib to II are sufficient as the extent of elective neck dissection for cN0 cases, taking into consideration the micrometastasis and regional recurrence. Levels Ib to III and Va may be sufficient to perform neck dissection for cN+ cases localized in the preauricular area or parotid gland nodes, considering lymphatic drainage [36]. Additional evidence is warranted to establish guidelines for cervical lymph node dissection.

#### 4.7.2. Parotidectomy

Tumors arising from the temporal bone often extend to the parotid gland through the Santorini fissure, foramen of Huschke, and the bone-cartilaginous junction of the external ear canal. Generally, the intraparotid lymph node is considered the first lymph node receiving drainage from the EAC. Lee et al. provided evidence regarding the use of elective parotidectomy for temporal bone carcinoma and recommended total parotidectomy for advanced SCC [37]. Parotidectomy is undoubtedly required in the surgical treatment of temporal bone SCC, and the extent of the resection of the parotid remains controversial [37,38,39].

#### 4.7.3. Manipulation of the Glenoid Fossa and TMJ

If the tumor extends anteriorly, manipulation of the glenoid fossa or TMJ is necessary. We selected the most appropriate procedure from several options, which included posterior capsulectomy, partial condylectomy, and total condylectomy after cutting the mandibular ramus or condylar neck. As previously mentioned, when control of the petrous carotid is required, surgeons should evaluate the feasibility of the transglenoid procedure.

### 4.8. Outcome of Temporal Bone Resection

Generally, advanced EAC-SCC cases have been considered as having poor prognosis. However, we revealed that only T4 status, not T3 status, was associated with poor prognosis [18]. Even for T4 cases, the en bloc resection with a negative surgical margin can improve the prognosis [16,17]. In the modified Pittsburgh classification system, T4 cases include the two types of cases that can be treated with either LTBR or STBR for curative resection. Yin et al. reported that most recurrent and metastatic patients died within two years [21] Our study showed the 2-year overall survival after temporal bone resection and revealed that the resection with negative surgical margin could offer a significantly better prognosis than the resection with positive surgical margin. Based on our findings, we believed that selecting an appropriate resection line for en bloc resection with a negative surgical margin can be associated with the improvement of patient prognosis.

### 4.9. Surgical Risks and Complications

STBR and extended LTBR has the risks of intra- and postoperative complications, such as petrous carotid injury, meningitis, cerebrospinal fluid leakage, lower cranial nerve palsy, occlusal interference, and sigmoid sinus thrombosis. During temporal bone resection, the surgeon should pay the most attention to avoiding injury to the petrous carotid during surgery.

## 5. Conclusions

With the development of a multidisciplinary approach, surgical techniques that were once challenging can now be performed more safely. Surgical approaches for advanced SCC of the temporal bone are diverse. They require accurate preoperative evaluation of the limits of anatomic tumor extension and preoperative consideration of the exact line of resection necessary to achieve negative margin resection. Increasing the control rate in case of positive margin resection of advanced cancer poses a challenge to surgeons. New treatment options, such as TPF-RT, have been reported. Consolidation of evidence from multiple institutions is warranted to develop appropriate treatment guidelines for advanced EAC-SCC.

## Figures and Tables

**Table 1:** Patient profiles.

Case	Sex	Age	Side	cT	cN	Pathology	Treatment	Folow-Up	Operation Time	Recurence
1	F	78	R	3	0	w-m SCC	Surgery	NED	8 h 10 m	
2	F	65	R	3	0	w-m SCC	CRT→Surgery	NED	10 h 32 m	
3	M	83	R	3	0	w SCC	Surgery *	DOD	9 h 19 m	T
4	F	66	R	3	0	w SCC	IC→Surgery	NED	6 h 13 m	
5	F	60	L	3	0	w SCC	IC→Surgery	NED	11 h 34 m	
6	F	69	L	3	1	w SCC	IC→CRT→Surgery	NED	17 h 29 m	
7	M	79	R	3	0	w SCC	Surgery *	DOC	10 h 1 m	
8	F	48	L	4	0	w SCC	IC→CRT→Surgery	NED	15 h 38 m	
9	F	60	R	4	0	w SCC	IC→Surgery *	NED	10 h 21 m	
10	M	65	L	3	1	w-m SCC	IC→Surgery *	NED	12 h 48 h	
11	F	71	R	4	3 b	w SCC	IC→Surgery *	NED	17 h 29 m	
12	F	66	R	3	1	w SCC	Surgery *	NED	10 h 41 m	
13	M	56	R	4	0	w SCC	IC→CRT→Surgery	AWD	20 h 30 m	T, M
14	F	33	R	4	0	w SCC	IC→Surgery *	AWD	17 h 57 m	N,M
15	F	57	R	4	0	w SCC	Surgery *	DOD	20 h 5 m	N
16	F	71	R	3	0	w-m SCC	Surgery *	NED	11 h 13 m	
17	F	71	R	4	0	w-m SCC	Surgery *	AWD	10 h 34 m	M
18	F	66	L	4	0	w SCC	Surgery *	NED	13 h 8 m	
19	M	78	R	4	0	w SCC	Surgery *	NED	20 h 59 m	
20	F	45	R	r4	0	w-m SCC	Surgery	DOD	18 h 10 m	N
21	M	66	R	r4	0	w SCC	Surgery	DOD	17 h 59 m	T

AWD, alive with disease; CRT, chemoradiotherapy; DOC, died of other cause; DOD, died of disease; F, female; I.C., induction chemotherapy; L, left; M, male/distant metastasis; N, cervical lymph node metastasis; NED, no evidence of disease; r, recurrent; R, right; SCC, squamous cell carcinoma; T, local recurrence; w, well-differentiated; w-m well-to-moderately differentiated. Asterisk indicates the addition of postoperative chemoradiotherapy.

**Table 2:** Detailed information of the surgical procedure.

Case	Craniotomy	Mast.	TGP	Type of Mandibulectomy	Enbloc Resection with			Ovening JF	Opening CC	Postoperative Paralysis	Margin	Parotidectomy	CLD	Dural Resection	Recon.
					SP	TMJ	Condyle								
1	−	+	−	Caps.	−	−	−	−	−	−		Partial	−	−	TM
2	−	+	+	Total	−	−	−	−	−	−		Superficial	II III IV	−	TM
3	−	+	−	Caps.	−	−	−	−	−	−	+	Superficial	II	−	TM
4	−	+	−	Caps.	−	−	−	−	−	−		Partial	−	−	TM
5	−	+	−	Caps.	−	−	−	−	−	−		Total	II III IV	−	ALT
6	Temporal	Limited	+	Total	+	−	−	Via mastoid	+	VII VIII		Total	II III	−	ALT
7	−	+	+	Total	−	−	−	−	−	Frontal branch of VII	+	Total	II III	−	TM
8	−	+	+	Total	+	−	+	Via mastoid	−	−		Total	II III	−	ALT
9	−	+	−	−	−	−	−	−	−	−		Total	IIa-b III	−	ALT
10	−	+	−	Caps.	−	−	−	−	−	−		Total	IIa-b III	−	ALT
11	−	+	−	Caps.	−	−	−	−	−	−		Total	I-V	−	ALT
12	−	+	−	−	−	−	−	−	−	−		Superficial	II III	−	ALT
13	Temporal	Limited	+	Total	+	−	−	Via mastoid	+	VII VIII	+	Total	II III IV	−	ALT
14	−	+	−	Caps.	+	−	−	−	−	−	+	Total	II-IV	−	ALT
15	Temporo-occipital	−	−	Total	+	+	+	Removal of JP	+	VII VIII	+	Total	I-IV	+	ALT
16	−	+	−	−	−	−	−	−	−	−		Superficial	II-III	−	ALT
17	−	+	−	Total	+	−	−	Via mastoid	−	VII		Total	I-V	−	ALT
18	−	+	−	Partial	+	−	+	−	−	−		Total	II-IV	−	ALT
19	Temporo-occipital	−	+	Total	+	−	−	Removal of JP	+	VII VIII		Total	Ib-V	+	ALT
20	Temporal	Limited	+	Angle unilateral free end mandibulectomy	+	−	−	Via mastoid	−	VII VIII	+	Total	II III IV	−	ALT
21	Temporal	Limited	+	Total	+	−	−	via mastoid	−	VII VIII	+	Superficial	II III	−	ALT

ALT; anterolateral thigh flap; Caps., posterior capsulectomy; CC, carotid canal; CLD, cervical lymph node dissection; JF, jugular foramen; JP, jugular process; Mast., mastoidectomy; Recon, reconstruction; SP, styloid process; TGP, transglenoid fossa procedure; TM, temporal muscle; TMJ, temporomandibular joint.

## Data Availability

The authors confirm that the data supporting the findings of this study are available within the article.
